# AlmaSDG: A Dataset of Scientific Articles' Contributions to the UN Sustainable Development Goals

**DOI:** 10.1038/s41597-026-07452-4

**Published:** 2026-06-09

**Authors:** Luca Bolognini, Elena Palmieri, Nicolò Donati, Giulia Grundler, Gianmarco Pappacoda, Federico Ruggeri, Andrea Galassi, Paolo Torroni

**Affiliations:** DISI, University of Bologna, Bologna, Italy

**Keywords:** Computer science, Information technology

## Abstract

The UN Agenda 2030 motivates the development of scalable methods and tools for assessing the contribution of research and higher education institutions to sustainable development. Current research efforts suffer from the lack of rigorous evaluation benchmarks. This paper describes a collaborative work with sustainability experts aimed at constructing AlmaSDG, the first pilot dataset of scientific articles labeled by multiple annotators along all SDGs. AlmaSDG is validated using inter-annotator agreement and inference of pre-trained models and tools.

## Background & Summary

### Motivation

The Sustainable Development Goals (SDGs) are a universal agenda for global sustainable development, adopted in 2015 by 193 states of the United Nations (UN), calling on all countries to adopt holistic development strategies for a sustainable advancement of the well-being of all citizens worldwide. SDGs, as can be seen in Table 1, address global challenges including poverty, inequality, climate change, environmental degradation, peace, and justice. The production and use of quality data is being increasingly recognized by the UN and its member States as an essential task for the assessment, monitoring and tracking of SDGs^1^. However, establishing clear target values is hampered by the broad scope of the goals themselves, their various scales (national, regional, global), the coverage of multiple issues in a single goal, and the ambiguous language of goal descriptions^2,3^.

**Table 1:** List of SDGs.

SDG Number	Description
1	End poverty in all its forms everywhere
2	End hunger, achieve food security and improved nutrition and promote sustainable agriculture
3	Ensure healthy lives and promote well-being for all at all ages
4	Ensure inclusive and equitable quality education and promote lifelong learning opportunities for all
5	Achieve gender equality and empower all women and girls
6	Ensure availability and sustainable management of water and sanitation for all
7	Ensure access to affordable, reliable, sustainable and modern energy for all
8	Promote sustained, inclusive and sustainable economic growth, full and productive employment and decent work for all
9	Build resilient infrastructure, promote inclusive and sustainable industrialization and foster innovation
10	Reduce inequality within and among countries
11	Make cities and human settlements inclusive, safe, resilient and sustainable
12	Ensure sustainable consumption and production patterns
13	Take urgent action to combat climate change and its impacts
14	Conserve and sustainably use the oceans, seas and marine resources for sustainable development
15	Protect, restore and promote sustainable use of terrestrial ecosystems, sustainably manage forests, combat desertification, and halt and reverse land degradation and halt biodiversity loss
16	Promote peaceful and inclusive societies for sustainable development, provide access to justice for all and build effective, accountable and inclusive institutions at all levels
17	Strengthen the means of implementation and revitalize the Global Partnership for Sustainable Development

Universities and research institutions are naturally interested in the agenda, not only from the perspective of quality education, which is one of the SDGs and a mission of universities but also because of the role they play in fostering scientific, technological, socio-cultural progress through research and dissemination. In recent years, the interest in measuring the contributions of research and higher education institutions towards the SDGs has grown considerably. Some important initiatives are Elsevier’s introduction of SDG indicators in Scopus^4^ and the Times Higher Education University Impact Rankings, also based on Scopus. Analyses have been conducted using bibliometric tools such as CiteSpace^5^.

Although these initiatives contribute to the discourse on SDGs, they suffer from the lack of a gold standard, which hampers the evaluation of these tools’ accuracy^6^.

To the best of our knowledge, there is no precise, shared understanding of what it means for a scientific article to contribute to an SDG. SDGs are defined broadly and their targets are highly interconnected, thereby progress towards one target is linked through complex feedbacks to other targets^2^. Moreover, most of the SDG progress metrics proposed in the literature are based on the SDGs’ target indicators, defined as measurable KPIs for monitoring advancements towards SDGs at the national or global level, not for evaluating the enabling research^7^. The assessment of the contribution of research and higher education institutions is instead primarily focused on student awareness and dedicated curricula, estimated through surveys involving experts^8^.

### Related Datasets

Existing SDG datasets vary by data format, data source, and labeling method.

For what concerns fully-automated labeling processes, Vanderfeesten^9^ uses the Aurora query system to retrieve about 1.4 million research publications from the Scopus database. These data are automatically labeled according to the queries without the supervision of human experts, thus possibly creating noisy data. LaFleur^10^ instead leverages ChatGPT to generate about 1,000 synthetic documents in different styles, such as newspaper articles and analytical reports. Their purpose is to improve a machine learning-based classifier providing further training data.

Vanderfeesten *et al*.^11^ build a human-labeled dataset by running a survey among researchers. Participants are asked to state whether specific publications were relevant for an SDG and to suggest other related publications. It is difficult to assess the reliability of this corpus: most samples are evaluated by only one researcher and there are several instances where the number of positive and negative evaluations is equal. Furthermore, most samples are evaluated only for one SDG, potentially creating false negatives with the remaining 16 SDGs.

The OSDG dataset^12^ is the result of the efforts of the OSDG Community Platform. It comprises textual paragraphs extracted from documents, such as policy briefs, reports, and scientific publications, annotated through crowd-sourcing. Each text comprises 3 to 6 sentences and is about 90 words on average. Each document is associated with a single label proposed by the source authors which is then propagated to every paragraph. The labels encompass SDG 1 to 16. Each paragraph is annotated by at least 3 volunteers, who accept or reject the proposed label. Every paragraph is kept in the dataset, whether its label is accepted or rejected, along with the number of rejections and acceptances. The resulting dataset comprises 41,689 single-label samples. This dataset has two main limitations: it does not consider SDG 17, and is constrained to a single label per paragraph, which may miss relevant contributions to multiple SDGs, creating false negatives.

Wulff and Meier^13^ propose a dataset (SKH) based on the SDG Knowledge Hub, which gathers news, guest articles, and policy briefs about Sustainable Development. Documents are labeled by their authors, who are assumed to be experts, considering all SDGs. Each document can be assigned multiple labels, which must then be confirmed by the platform editors. However, using authors as annotators of their own articles may introduce bias.

Yao *et al*.^14^ curate a dataset of 900 lecture video descriptions taken from SDG Academy, a platform that contains several Open Educational Resources (OERs). Each description is in English, at least 50 words, associated with a minimum of one and a maximum of four SGDs. We could not find details on the data annotation process.

Table 2 summarizes the main characteristics of publicly available datasets.

**Table 2:** Summary of existing datasets related to SDGs.

Dataset	Domain	Labelling	Annotation method	# Samples
Aurora Queries^9^	Scientific Publications	Multi Label	Automatic: Query	1.4m
Aurora Survey^11^	Scientific Publications	Mostly Single Label	Crowdsourced	10,682
OSDG^12^	Policy Briefs, Reports, Scientific Publications	Single Label	Crowdsourced	41,689
SDG Knowledge Hub^13^	News, Guest Articles, Policy Briefs	Multi Label	Author Proposed	9,172
SDG Academy^14^	Video lectures	Multi Label	Author Proposed	900
AlmaSDG (**Ours**)	Scientific Publications	Multi Label	Annotation with guidelines	139

### Contribution

We propose AlmaSDG: a multi-label dataset manually annotated by human experts. Together with AlmaSDG, we provide a set of guidelines that define what it means for a scientific article to contribute to an SDG. The dataset comprises 139 scientific articles annotated according to all 17 SDGs, for a total of 2,363 labels. We validate AlmaSDG by Inter-Annotator Agreement (IAA) measures and by running an extensive experimentation with existing SDG classification tools and selected Large Language Models (LLMs). To the best of our knowledge, AlmaSDG is the first pilot dataset for method development labeled by multiple annotators that follow a set of specific guidelines, and for which a measure of IAA is provided.

## Methods

AlmaSDG is the result of a 12-month-long joint effort of stakeholders, academic experts in SDGs, and computer scientist, comprising an iterative process of guidelines creation and data labeling.

### Guidelines Definition

Defining what is a contribution towards the SDGs is complicated by the wide scope, interrelations and ambiguity of the SDG descriptions^15^. We run a first pilot study to explore the possibility of using the SDGs’ targets as guidelines. We observe that some SDGs, such as 10 (reduced inequalities) and 3 (good health and well-being), are difficult to capture using the targets. Targets are often too specific and fail to represent the multi-faceted scope of the SDG. They are also open to subjective interpretation. This highlights the inadequacy, for annotation purposes, of general descriptions of the SDG aims and scope, and of lists of SDG targets, hence the need to provide annotators with fine-grained instructions.

For this reason, we adopt a *prescriptive* approach^16^, which aims to stimulate a shared belief among annotators through the definition of reliable and unambiguous guidelines. We develop a novel set of guidelines for annotating SDGs in scientific publications. To do so, we collaborate with 5 academic experts in sustainability and sustainable development, social inclusion and inclusive education, renewable energy, management and sustainable valorization of food waste, circular economy and urban mining, ecodesign for urban resilience, technologies for developing countries, and water supply. One of these experts is a university representative at the UN and the European Commission. We focus on two main aspects: first, we define what constitutes a research contribution advancing the SDG’s progress, then, we describe the themes addressed by each SDG.

Following Armitage *et al*.^15^, we differentiate between *direct* and *indirect* contribution to a goal or a target. A direct contribution either studies, develops, or applies methods and technologies. An indirect contribution lays the groundwork for future developments with a specific proposal, such as reviews of the state of the art, evaluation methods, or data gathering. We make the case that this distinction is necessary because if we consider only the goals’ targets, the annotation process may neglect less tangible but equally important contributions. At the same time, it is important to distinguish between actual (indirect) contributions and cursory references to SDG topics without an actual contribution.

As a second step, we consider both the SDG thematic areas and the UN targets, to create a detailed list of contribution areas specific to each SDG, for a total of 165 items. Moreover, we include examples of positive and negative cases to provide references. Such examples were proposed by the experts based on their knowledge or on ambiguous cases found during the pilot study.

### Data Annotation

The SDG experts in our team confirm that major research databases, such as Scopus and PubMed represent research disciplines unevenly, by favoring publications in scientific areas over humanities^17^. Considering that as a limiting factor, we adopt a comprehensive publication repository of the University of Bologna as a data source. We select only articles whose metadata is publicly available. The data source does not only contain SDG-relevant publications. A random sampling would thus result in a large majority of negative samples. Therefore, we proceed by querying the data source using relevant fragments from SDG descriptors as search keywords. We then select 139 articles that appear potentially relevant to SDGs, trying to cover all SDGs.

We adopt a three-fold annotation process. We first annotate the corpus, then measure the Inter-Annotator Agreement (IAA), and finally solve disagreements. Regarding the annotation, we form a multi-disciplinary group of 8 NLP researchers (henceforth, workers) with English proficiency, 4 of whom with a track record in corpus creation.We pair up workers in 4 groups. Each group labels the whole dataset focusing on a subset of 4/5 SDGs. We provide workers with relevant metadata for each publication, specifically *title*, *abstract*, and *keywords*. We do not consider the full text.Each worker independently annotates the 139 documents as contributing or not contributing to each assigned SDG.In the final step, workers discuss and solve disagreements to assign the final labels to each paper. When this is not possible, additional workers are involved to discuss and converge.

## Data Record

AlmaSDG^18^ is publicly available on the Zenodo platform: https://zenodo.org/records/19606053. The repository is organized as follows:

- File alma_sdg.csv. It contains the data in CSV format. Each row is a data point.
- File guidelines.pdf. It contains the annotation guidelines.
- Files Annotator0.xlsx, …, Annotator7.xlsx. Each file contains the labels assigned to each data point by one annotator.
- File copyright.csv. It reports copyright information.

The data points in the CSV file are structured as follows:

- **Metadata**: handle (the article identifier), title, abstract, and keywords;
- **Labels**: 17 labels with binary values True or False, one per SDG.

It is possible to retrieve each article’s metadata from the University of Bologna’s publication repository (http://cris.unibo.it) using the article identifier (handle) as part of the web address. For example, if the identifier is 11585/956123, the metadata are available at https://cris.unibo.it/handle/11585/956123.

The dataset comprises 2,363 data points associated with 139 articles. Table 3 shows label statistics. The distribution of positive labels varies across SDGs, with SDG 10 (reduced inequality) exhibiting the highest number of positive instances (32), followed by SDG 12 (responsible consumption and production) and 3 (good health and well-being). On the other hand, SDG 1 (no poverty), 14 (life below water), 17 (partnerships for the goals), 6 (clean water and sanitation), and 15 (life on land) have less than 10 positive instances, which reflects the difficulty in retrieving an even amount of articles. Table 4 shows that only 13 articles out of 139 do not contribute to any SDGs, whereas about half of the articles contribute to multiple SDGs.

**Table 3:** AlmaSDG corpus statistics on positive labels.

SDG	1	2	3	4	5	6	7	8	9	10	11	12	13	14	15	16	17	Avg
# Positive	5	12	25	10	13	8	10	15	13	32	23	25	17	7	9	11	7	14.24
% Positive	3.6	8.6	18.0	7.2	9.4	5.8	7.2	10.8	9.4	23.0	16.5	18.0	12.2	5.0	6.5	7.9	5.0	10.24

**Table 4:** Number of labels associated with each document.

# of labels per document	0	1	2	3	4+
# of documents	13	57	39	19	11

## Technical Validation

We primarily validate our work by measuring the agreement between human annotators. Moreover, we evaluate the performances of 3 LLMs and 4 state-of-the-art methods on our dataset.

### Inter-Annotator Agreement

We measure the IAA for each SDG using Cohen’s Kappa^19^. The results are shown in Table 5, along with the corresponding annotator pairs, labeled as G1, …, G4. IAA is SDG-dependent, with the same pair of workers exhibiting different levels of agreement in different SDGs. For SDG 5 (gender equality), we reach the highest agreement score, 0.86, corresponding to “almost perfect agreement”^20^. Annotating other SDGs is a more challenging task. For example, for 8 (decent work and economic growth) and 16 (peace, justice and strong institutions), we obtain only “fair agreement”, with IAA scores of 0.23 and 0.24. SDG 5 and its associated targets are relatively straightforward to identify because gender equality is a recurrent issue in daily life, and is often explicitly stated as one of the goals of the publication. In contrast, SDGs 8 and 16 have a much broader and complex scope, making the annotation process more challenging and subjective. Overall, we achieve an average IAA of 0.54, corresponding to “moderate agreement”.

**Table 5:** Agreement between human annotators. For each SDG, we report the IAA and the annotator group.

SDG	1	2	3	4	5	6	7	8	9	10	11	12	13	14	15	16	17	Avg
IAA	0.44	0.39	0.60	0.57	0.86	0.46	0.76	0.23	0.27	0.59	0.53	0.65	0.62	0.76	0.74	0.24	0.49	0.54
Group	G1	G1	G1	G2	G2	G3	G3	G1	G3	G2	G3	G4	G4	G4	G4	G2	G4	

### Experimental Evaluation

**Fig. 1:**
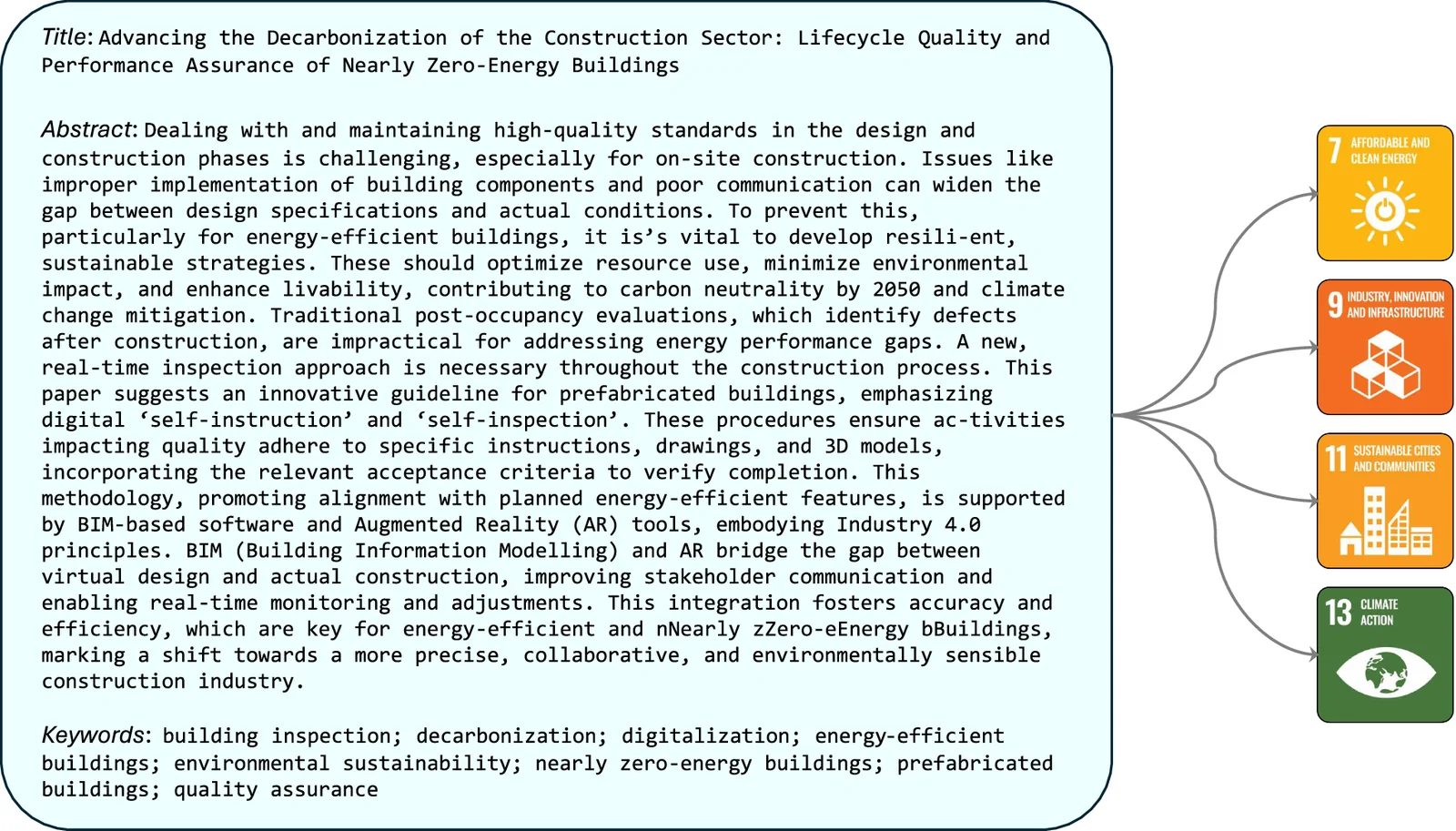
Example of AlmaSDG input document and labels.

We evaluate the inference of pre-trained models and tools over our dataset. We frame the problem as a multi-label document classification task. An input document consists of the title, abstract, and keywords of a scientific article. Each binary label indicates the contribution of the scientific article to a specific SDG (see Figure 1). We report results in terms of F1 score on each SDG and macro-averaged Precision, Recall, and F1 score.

As LLMs, we consider GPT, Gemini, and Llama. We set up a zero-shot inference setting designed to imitate the human annotation process. First, we design a prompt template that is based on our guidelines. Then, for each document, we instantiate the template with the same metadata used by humans during the annotation. To ensure a deterministic classification setting, we set the temperature to 0.

We compare the results of GPT, Gemini, and Llama against three available SDG classification tools, which we selected because they are publicly available and accessible by API:

- **Aurora**^21^: the classifier trained on Aurora queries dataset^9^. It consists of several binary BERT-based classifiers, one for each SDG.
- **Elsevier**^22^: a BERT-based classifier trained on data retrieved through Elsevier queries^23^. The classifier is designed to output a probability distribution over all the SDGs except for SDG 17, and then select a single label. In the tables, this setting is named “Elsevier-S”, for single-label. However, since we frame the problem as a multi-label classification, we consider an additional configuration by applying an arbitrary threshold of 0.1 to determine positive labels. In the tables, this setting is named “Elsevier-M”, for multi-label.
- **OSDG**^24^: the hybrid tool developed by the OSDG project, which relies on ontologies and machine learning methods. For each instance, the tool can assign at most 3 SDGs. We shall remark that the tool’s output was sometimes inconsistent, as consecutive queries yielded different results. We consider the July 2024 version of the tool.

We report aggregate and detailed results in Tables 6 and 7. We observe that GPT yields the best macro F1 score (0.68), followed by Gemini (0.63). In contrast, Llama is the least performing LLM by a notable margin (0.48). Regarding existing tools, Elsevier-M reaches a score of 0.54, while Elsevier-S is more precise but less sensitive, resulting in a lower macro F1 (0.44). Similarly, OSDG reports high precision but lower recall than Elsevier-M, achieving an F1 macro score of 0.49. Aurora is the worst-performing model with an F1 macro score of 0.35, significantly lower than other competitors. Overall, GPT, OSDG, Elsevier-S, and Aurora have higher precision than recall, whereas Gemini, Llama, and Elsevier-M have better recall. Looking at the F1 score on each SDG, we observe that LLMs report specialized performance on certain SDGs. For instance, Gemini notably outperforms all competitors on SDG 5 (0.96 F1 score). GPT is the top-performing model on several SDGs, among which is SDG 17, which is one of the hardest ones based on the low support (i.e., the number of positive samples). In contrast, all existing tools like Aurora, Elsevier, and OSDG either fail or are not designed to classify any sample associated with SDG 17. In several cases, the classifiers’ performance is aligned with the human IAA, performing worse on the SDGs that are more difficult to annotate, such as SDGs 8 and 9. Similarly, half the models yield an F1 score of at least 0.80 on SDG 5, which has the highest IAA.

**Table 6:** Experimental evaluation as macro-averaged precision, recall, and F1 score.

Tool	Macro Precision	Macro Recall	Macro F1
Gemini	0.54	0.81	0.63
GPT	0.78	0.66	**0.68**
Llama	0.36	0.87	0.48
Aurora	0.63	0.28	0.35
Elsevier-S	0.60	0.40	0.44
Elsevier-M	0.54	0.66	0.54
OSDG	0.60	0.46	0.49

**Table 7:** Experimental evaluation as F1 score on each SDG.

Tool	SDG																
	1	2	3	4	5	6	7	8	9	10	11	12	13	14	15	16	17
Gemini	0.43	**0.67**	0.64	0.71	**0.96**	0.73	0.74	**0.51**	0.55	**0.63**	0.69	0.78	0.58	0.82	0.46	0.38	0.44
GPT	**0.50**	**0.67**	**0.83**	0.75	0.63	**0.88**	**0.78**	0.42	**0.61**	0.58	**0.71**	**0.84**	0.74	**0.86**	0.80	0.44	**0.60**
Llama	0.28	0.59	0.56	0.44	0.80	0.67	0.50	0.29	0.37	0.56	0.64	0.73	0.45	0.56	0.26	0.25	0.16
Aurora	**0.50**	0.27	0.28	0.43	0.67	0.67	0.40	0.30	0.00	0.36	0.20	0.21	0.26	0.33	0.62	0.40	0.00
Elsevier-S	0.36	0.40	0.28	0.67	0.60	0.71	0.40	0.46	0.00	0.27	0.47	0.51	0.50	0.50	**0.82**	0.48	0.00
Elsevier-M	0.21	0.64	0.33	**0.78**	0.93	0.67	0.47	0.41	0.41	0.54	0.59	0.56	0.60	0.67	0.89	**0.55**	0.00
OSDG	0.32	0.44	0.65	0.75	0.87	0.70	0.59	0.29	0.12	0.31	0.42	0.40	**0.75**	0.46	0.67	**0.55**	0.00

## Data Availability

AlmaSDG^18^ is publicly available on the Zenodo platform: https://zenodo.org/records/19606053.

## Code availability

The code used to compute the agreement and run the experiments, including the prompts, is publicly available, alongside the annotated dataset and guidelines, on the GitHub repository: https://github.com/nlp-unibo/AlmaSDG. All the code is inside the SCRIPTS folder. The experimental results are in the VALIDATION_RESULTS folder.

As LLMs, we use GPT-4o-mini, gemini-1.5-flash-001, and Llama3-8B-Instruct.
